# The development and evaluation of a computerized decision aid for the treatment of psychotic disorders

**DOI:** 10.1186/s12888-018-1750-7

**Published:** 2018-06-01

**Authors:** Magda Tasma, Lukas O. Roebroek, Edith J. Liemburg, Henderikus Knegtering, Philippe A. Delespaul, Albert Boonstra, Marte Swart, Stynke Castelein

**Affiliations:** 1 Lentis Psychiatric Institute, Lentis Research, Hereweg 80, 9725 AG Groningen, The Netherlands; 2 Rob Giel Research Centre, University of Groningen, University Medical Centre Groningen, Groningen, The Netherlands; 3 Faculty of Economics and Business, University of Groningen, Groningen, The Netherlands; 4 Faculty of Psychiatry & Psychology, Maastricht University, Maastricht, The Netherlands; 5 Mondriaan Mental Health Trust, Heerlen-Maastricht, The Netherlands; 6 Faculty of Behavioural and Social Sciences, University of Groningen, Groningen, The Netherlands

**Keywords:** Clinical decision aid, Psychotic disorder, Guidelines, Routine outcome monitoring, Optimal treatment, Treatment recommendations

## Abstract

**Background:**

Routinely monitoring of symptoms and medical needs can improve the diagnostics and treatment of medical problems, including psychiatric. However, several studies show that few clinicians use Routine Outcome Monitoring (ROM) in their daily work. We describe the development and first evaluation of a ROM based computerized clinical decision aid, Treatment-E-Assist (TREAT) for the treatment of psychotic disorders. The goal is to generate personalized treatment recommendations, based on international guidelines combined with outcomes of mental and physical health acquired through ROM. We present a pilot study aimed to assess the feasibility of this computerized clinical decision aid in daily clinical practice by evaluating clinicians’ experiences with the system.

**Methods:**

Clinical decision algorithms were developed based on international schizophrenia treatment guidelines and the input of multidisciplinary expert panels from multiple psychiatric institutes. Yearly obtained diagnostic (ROM) information of patients was presented to treating clinicians combined with treatment suggestions generated by the algorithms of TREAT. In this pilot study 6 clinicians and 16 patients of Lentis Psychiatric Institute used the application. Clinicians were interviewed and asked to fill out self-report questionnaires evaluating their opinions about ROM and the effectiveness of TREAT.

**Results:**

Six clinicians and 16 patients with psychotic disorders participated in the pilot study. The clinicians were psychiatrists, physicians and nurse-practitioners which all worked at least 8 years in mental health care of which at least 3 years treating patients with psychotic illnesses. All Clinicians found TREAT easy to use and would like to continue using the application. They reported that TREAT offered support in using diagnostic ROM information when drafting the treatment plans, by creating more awareness of current treatment options.

**Conclusion:**

This article presents a pilot study on the implementation of a computerized clinical decision aid linking routine outcome monitoring to clinical guidelines in order to generate personalized treatment advice. TREAT was found to be feasible for daily clinical practice and effective based on this first evaluation by clinicians. However, adjustments have to be made to the system and algorithms of the application. The ultimate goal is to provide appropriate evidence based care for patients with severe mental illnesses.

**Electronic supplementary material:**

The online version of this article (10.1186/s12888-018-1750-7) contains supplementary material, which is available to authorized users.

## Background

### Treatment of psychotic disorders

Almost 1 % of the population in the western world will eventually fulfil the criteria of schizophrenia or a related severe mental illness [1]. Core symptoms of many people suffering from psychotic disorders are hallucinations, delusions, incoherent thoughts, memory problems, loss of initiative, flat affect, poverty of speech and social withdrawal [2]. Moreover, patients frequently experience problems with psychosocial functioning, such as a lack of daytime activities, social contacts, intimate relationships and a reduced quality of life [3, 4]. They often have poor physical health and experience medication side effects that contribute to an early onset of cardiovascular diseases. Different studies have shown a reduced life expectancy ranging from 10 up to 28 years [5, 6]. Some patients manage to recover both in terms of their symptoms, as well as in reaching personal and social goals. However the majority only partially recovers, with recurrence of symptoms and enduring personal and social problems often for the rest of their life. Especially patients with the most severe symptoms (fulfilling criteria for schizophrenia or schizoaffective disorders) often need lifetime medical, psychiatric and social care. Recommended treatment options are described in national treatment guidelines; in the Netherlands the Multidisciplinary Guideline for Schizophrenia is used (which is largely in line with the NICE guideline) [7]. The Optimal Treatment Project revealed that 2 years of optimal, evidence-based treatment led towards a clear trend in recovery from clinical impairment and social disability of patients with psychotic disorders [8]. Despite increasing evidence that pharmacological and psychosocial interventions are effective in improving clinical symptoms and patients’ functioning, the availability of treatment interventions and integration in psychiatric care is often suboptimal [9]. Also, many patients with psychotic disorders find it difficult to express their needs, show a decreased awareness of their symptoms and only partially understand the different possible treatment options. Therefore, psychological, medical and social problems often go undetected or untreated [10]. There is a challenge to monitor symptoms and unmet care needs of these patients in order to offer optimal care, especially in realizing their varying needs in different domains for many years.

### Routine outcome monitoring

Routine outcome monitoring (ROM) is one such way to monitor symptoms and care needs. ROM can be described as the use of standardized instruments to systematically and repeatedly measure different aspects of patients’ symptoms, health, social functioning and wellbeing in order to improve their treatment [11, 12]. For patients with schizophrenia and related mental health problems, regular participation in ROM contributes to systematic evaluation of their varying needs in (mental) health care over many years. This could offer these patients relevant treatment options adjusted to actual needs. However, only few clinicians use ROM data in their day-to-day work [13, 14]. In the Northern Netherlands, an extensive ROM screening called the Pharmacotherapy Monitoring and Outcome Survey (ROM-Phamous), consisting of a large array of instruments [15], has been implemented since 2007. Its main target is to identify needs of care in psychiatric, medical and social domains in order to optimize the treatment of patients with psychotic disorders. The data obtained also allows for scientific research. Although it has been shown that ROM-Phamous successfully helps to identify unmet needs, it is still not optimally used in clinical decision making and in offering recommended evidence-based treatment options to people with psychotic disorders [16–18].

### Treatment E-assist

There is often not just a single best option when making treatment decisions in healthcare. Different treatment options may have varying risks and benefits, making it challenging to offer the optimal option when decisions are sensitive to personal preference. Clinical decision aids (CDAs) are evidence-based tools to support decision making in healthcare and have been gaining popularity in various medical disciplines [19]. A recent meta-analysis shows CDAs improve patients’ knowledge about available treatment options, facilitate accurate risk perception and increase their active involvement in the decision making process [20]. While knowledge about effective mental health care keeps growing, translation to daily clinical practice is lagging [21, 22]. CDAs can serve as a guideline implementation strategy by transferring evidence-based knowledge to day-to-day patient care. Despite the potential benefits of CDAs, their use in mental healthcare is very limited. In one study, a computerized CDA linking patient specific data to guidelines led to a decrease of symptoms and lowered re-hospitalization rates among people with psychotic disorders [23]. However, all diagnostic measurements for this CDA had to be collected by clinicians themselves, making the process time consuming. In the current study we describe Treatment E-Assist (TREAT). This is a recently developed computerized CDA that combines diagnostic patient data, collected using ROM-Phamous, with guidelines. TREAT facilitates the use of ROM-Phamous in daily clinical practice by summarising patients’ unmet needs. As a second step, evidence-based treatment recommendations based on the Multidisciplinary Guideline for Schizophrenia are generated to assist the clinician and patient to make shared decisions about these unmet needs.

### Research aim

This article describes the development of TREAT and the results of the pilot study, evaluating TREAT, as a computerized CDA designed for the treatment of patients with psychotic disorders. The pilot study tests the feasibility of TREAT in daily clinical practice by evaluating clinicians’ experiences when working with the application.

## Methods

### Development of TREAT

#### Substantive design

The algorithms of TREAT are based on ROM-Phamous data and the Multidisciplinary Guideline for Schizophrenia [7]. The algorithms were designed in collaboration with two multidisciplinary expert panels, both consisting of 7 members representing different institutions. These panels included researchers, psychiatrists, psychologists and nurse-practitioners, all experienced in working with ROM-Phamous. The first panel focused on somatic problems and pharmacotherapy and the second panel focused on psychosocial interventions. Participants from the expert panels did not participate in the pilot study described hereafter.

**Fig. 1:**
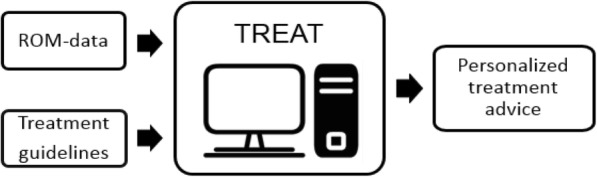
Schematic of TREAT

The first session of the expert panels was an introduction of the project and a brainstorm to collect first ideas and thoughts. Next, researchers proposed possible problematic domains in care for people with a psychotic disorder: positive symptoms, negative symptoms, cognitive symptoms, psychosocial problems, and somatic problems. Each domain was further divided into subcategories. The researchers selected matching items from the ROM-Phamous instruments for each subcategory and proposed cut-off scores. TREAT displays these subcategories as problematic when patient measures on the matching instruments exceed the cut-off scores. The draft proposal was then discussed with the panels, until consensus was reached. Cut-off scores were based on expert opinions when explicit guidelines were lacking. Instead of the Multidisciplinary Guideline for Schizophrenia [7], the pharmacotherapy panel decided to use a more detailed guideline for the treatment of cardiovascular risk factors based on the input of somatic doctors working in psychiatry (of Mental Health Care Center Drenthe, The Netherlands). This guideline (Guideline for Cardiovascular Risk Management Drenthe) offers cut-off scores and treatment recommendations on cardiovascular risk factors for patients who have been using antipsychotic medication for a long period of time. Finally, the researchers proposed treatment recommendations to both panels for each care domain, based on the Multidisciplinary Guideline for Schizophrenia [7]. Treatment recommendations were complemented where necessary and discussed until consensus was reached. The final TREAT algorithms were discussed with two guideline experts (HK and SC) to assess whether the Multidisciplinary Guideline for Schizophrenia [7], had been properly followed. Figure 1 depicts a schematic of TREAT.

#### Program design

**Fig. 2:**
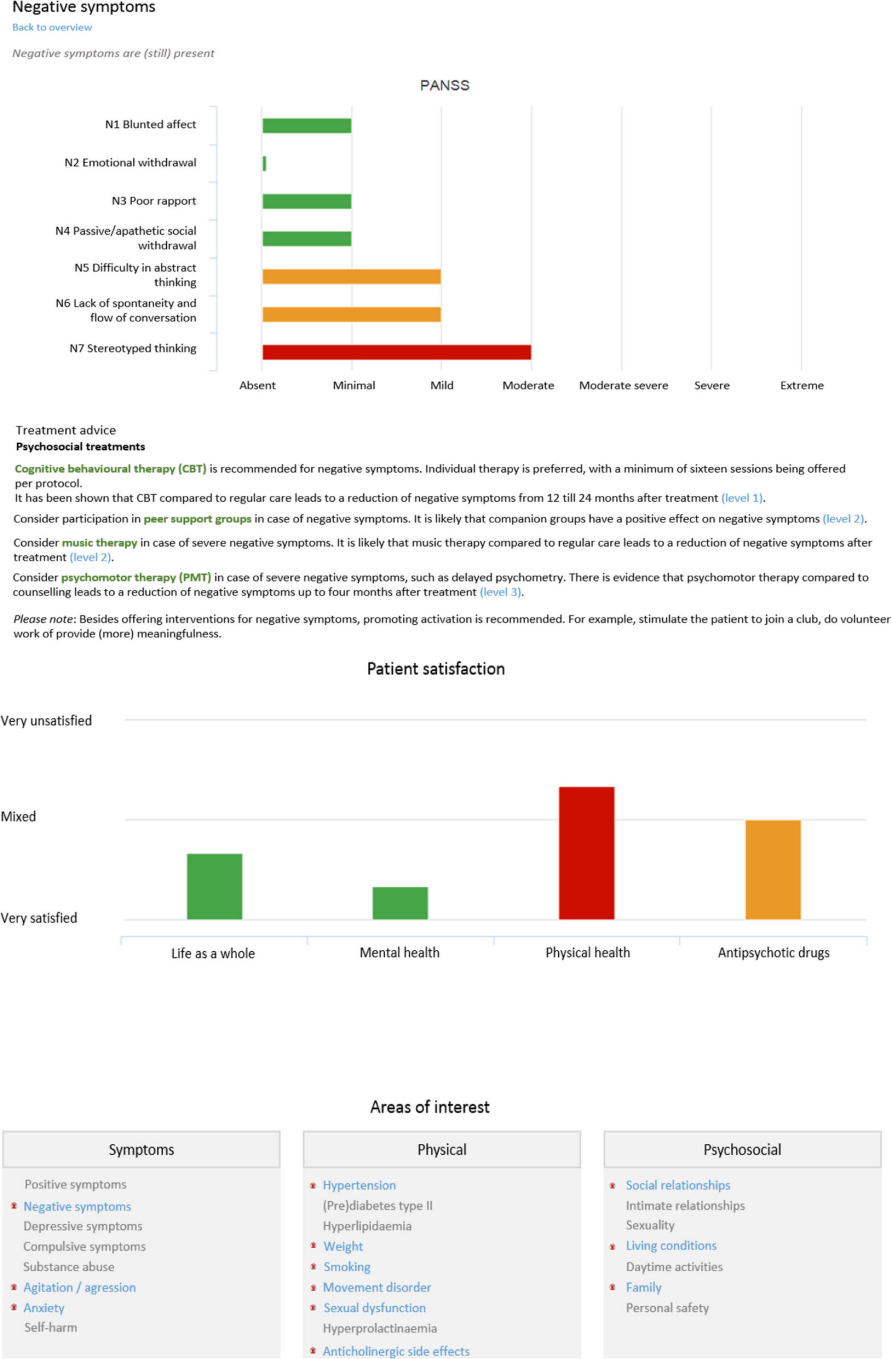
Screenshot of TREAT 1

**Fig. 3:**
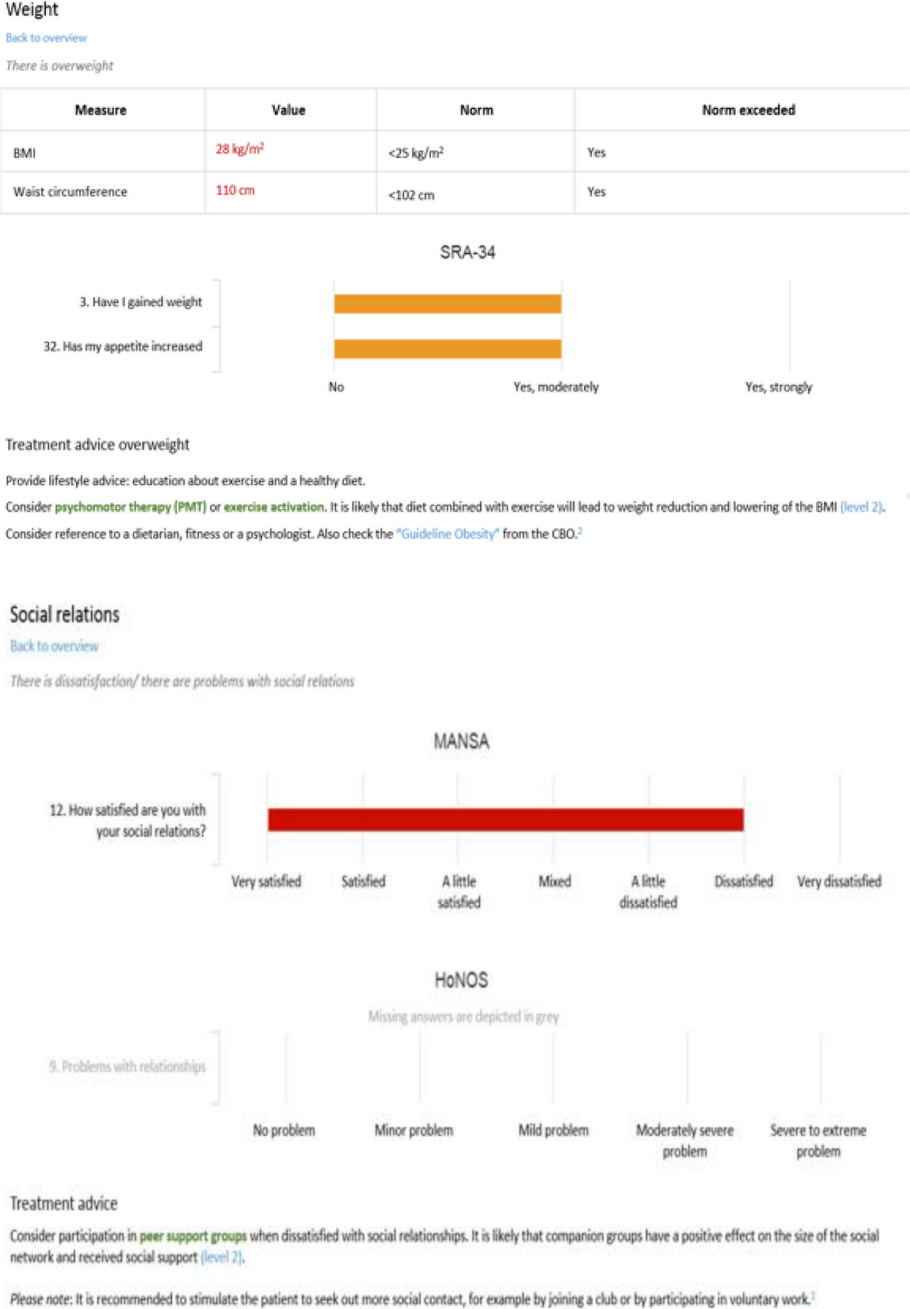
Screenshot of TREAT 2

ROM-Phamous and TREAT were both developed by a company (RoQua) that specializes in privacy protecting ROM systems that are accessed via the electronic patient record. TREAT has been built as an addition to the ROM-Phamous system and generates an interactive (Dutch) report for each individual patient. The first part of the report displays a summary of the ROM results. It contains a graph depicting symptom dimensions, treatment effects, patient satisfaction and unmet needs in different areas of life, such as mental health, physical health and antipsychotic medication. It also depicts general information about the patient, for instance the types of treatment the patient has received during the previous year. Finally, an overview is depicted of all care domains that are measured by ROM highlighting (in blue) which areas might (still) be problematic for a patient as depicted in Fig. 2. When a clinician selects a care domain, the relevant instruments, items, scores and treatment recommendations are displayed. In the graphs, the color of a bar indicates the severity of the problem (green = no problem, orange = potential problem and red = problem). On each page that depicts a care domain, one can click on the “back to overview” button to move back to the summary page. Clinicians can navigate through the TREAT report to assess all relevant ROM-Phamous results and treatment recommendations of a patient as depicted in Figs. 2 and 3.

### Pilot study

#### Participants

Six clinicians and sixteen patients of two (outpatient) Functional Assertive Community Treatment (FACT) teams of Lentis Psychiatric Institute participated in the pilot study. The clinicians were three psychiatrists, two nurse-practitioners and one physician. Patients were eligible for the study when they had a DSM IV diagnosis on the psychosis spectrum, or a personality or mood disorder with psychotic features. All patients filled out an informed consent form. The procedures were in accordance with the declaration of Helsinki as confirmed by the Medical Ethics Committee of the University Medical Center Groningen.

#### Procedure

Before the clinicians started using TREAT, they were asked to fill out a questionnaire that assessed their opinions about ROM-Phamous. Next, each clinician used TREAT before and/or during the discussion of the ROM results with a patient. The first three patients with psychotic disorders scheduled to have an appointment with their clinician to discuss their yearly ROM results, were asked to participate in the study. One of the six clinicians only participated with one patient due to planning issues during the time of this pilot study and patients declining to participate. The nurse who performed the ROM-Phamous screening was instructed to create the individual TREAT report. Both the nurses and the clinicians received instructions about TREAT from a researcher (MT). During these hour long treatment sessions, ROM results are discussed and treatment plans are drafted or adjusted. Afterwards, clinicians and patients filled out a questionnaire about the clinical decision making process during the sessions. This included questions about the topics that were discussed and the treatment options that were considered. At the end of the pilot study, clinicians filled out a questionnaire and participated in a brief open interview that both assessed their experiences with TREAT.

#### Measures

Assessments were made with two self-developed, theory-based Dutch questionnaires. The first one was the ‘ROM State-of-Mind’ consisting of 24 items, with the first 22 items assessing participants’ acceptance of ROM-Phamous on a Likert scale from 1 (completely disagree) to 5 (completely agree). Item 23 assessed overall appreciation of ROM-Phamous on a scale from 1 (very poorly) to 10 (excellent) and item 24 was an open question about suggestions and comments regarding ROM-Phamous. Items constituted seven subscales, of which five had a high internal consistency (Cronbach’s Alpha ≥ .7) and two a low internal consistency (Cronbach’s Alpha < .4) [18].

Secondly, participants filled out the ‘TREAT State-of-Mind’ consisting of 27 items assessing statements about TREAT, each rated on a Likert scale from 1 (completely disagree) to 5 (completely agree). The items constituted eight subscales, measuring usage behaviour, support, power, issue-impact, emotion, ease of use, usefulness, and facilitating conditions. The following two items assessed the acceptance of TREAT in general and the integration of both TREAT and ROM-Phamous. Both items were rated on a scale from 1 (very poorly) to 10 (excellent). The next item was an open question to collect ideas, comments or suggestions about TREAT.

The questionnaire also registered the amount of times clinicians consulted TREAT and for how long they had used TREAT on average per consult. The remaining six items assessed clinicians’ characteristics: profession, department, gender, age, and number of years working in mental health care and psychosis care.

We used the Clinical Decision Making in Routine Care (CDRC) questionnaire [24], to assess the treatment sessions. This questionnaire was translated in to Dutch and expanded with specific categories, largely representing the problems that are assessed with TREAT (see Additional file 1). The CDRC has a staff and patient version, consisting of 22 and 21 items each. The original authors, CEDAR study group, provided permission to use the questionnaire.

## Results

### Clinicians’ experiences with ROM and TREAT

All clinicians had worked with ROM-Phamous for 7 years on average (ranging from 3 years to 9 years).

They were generally positive about ROM-Phamous, grading it with an average of 7 (SD 0.89) on a scale from 1 (poor) to 10 (excellent). They have positive feelings towards ROM-Phamous (subscale ‘emotion’), see it as a useful addition to their job (subscale ‘usefulness’), but don’t find it very easy to use (subscale ‘ease of use’). Results are depicted in Table 1. Clinicians were also positive about TREAT with an average rating of 7.5 (SD 0.84) and the integration of TREAT and ROM with an average rating of 7.3 (SD 1.37) on a scale from 1 (poor) to 10 (excellent). Most clinicians had used TREAT at least three times during the pilot study, with the exception of one clinician who only worked with TREAT a single time. The time TREAT was used differed per user: 5 to 15 min (1 psychiatrist), 15 to 30 min (1 nurse-practitioner and 1 physician) and more than 30 min per session (2 psychiatrist and 1 nurse-practitioner). Most clinicians find TREAT useful and expect it will help them improve their work. They state that it helps to interpret the (diagnostical) ROM-Phamous results and offers support in drafting the treatment plan. It also enhances awareness of the existing treatment options (subscale ‘usefulness’). TREAT fits with good clinical care, clinicians are proud of its development (subscale ‘emotion’) and expect to use TREAT in the future (subscale ‘usage behaviour’). TREAT is thought to be easy to use and requires little mental effort. Opinions about the lay-out of TREAT are mixed (subscale ‘ease of use’), for example some clinicians preferred the graphs displaying data vertically whilst others preferred it horizontally. Most clinicians state that they have enough time to use TREAT in daily clinical practice and that it helps them to work more efficiently (subscale ‘facilitating conditions’). They do not think TREAT will have impact on their professional autonomy (subscale ‘power’), on their job or on patient care in general (subscale ‘issue-impact’). Results are depicted in Table 2. Some clinicians preferred to use TREAT on the computer while others preferred to print the information. The topics that were mentioned in the open interview are depicted in Table 3.

**Table 1:** Scores on the ROM State-of-Mind questionnaire (clinicians)

Subscale	Items	Mean Score (SD)
Acceptance	2. I use ROM-Phamous results in the treatment of my patients.	4,00 (0,63)
	22. I actively use the information offered by ROM-Phamous.	3,00 (0,63)
Support	1. I express my concerns about ROM.	3,00 (1,27)
	21. I tell people that it’s good that ROM-Phamous exists.	3,50 (1,52)
Power	13. I experience ROM-Phamous as a form of behavioural control.	1,67 (0,52)
	18. Because of ROM-Phamous I have more control over my job.	2,83 (0,75)
Emotion	5. Use of ROM-Phamous fits with my professional values and beliefs.	4,00 (0,89)
	6. Use of ROM-Phamous fits with good clinical care.	4,67 (0,52)
	7. I am proud that ROM-Phamous is used in my institution.	3,67 (0,82)
	8. I am worried about the existence of ROM-Phamous.	2,17 (1,47)
Ease of use	3. ROM-Phamous results are easy to interpret.	3,00 (0,89)
	9. ROM-Phamous is easy to use.	2,83 (0,75)
	10. Working with ROM-Phamous requires little (extra) mental effort.	2,67 (0,82)
Usefulness	4. ROM-Phamous adds value to the treatment of my patients.	4,33 (0,52)
	11. Because of ROM-Phamous I am better able to perform my job.	3,67 (0,82)
	12. Because of ROM-Phamous I am better supported in my job.	4,17 (0,75)
	15. The instruments of the ROM-Phamous protocol provide me with enough valuable information about my patients.	3,83 (0,41)
	16. ROM-Phamous identifies care needs.	4,00 (0,63)
	17. Because of ROM-Phamous more thought goes into care modules.	3,50 (0,55)
Facilitating conditions	14. I have enough time to use ROM-Phamous in my daily work.	2,00 (0,63)
	19. Because of ROM-Phamous I am able to work more efficiently.	3,50 (0,55)
	20. Using ROM-Phamous costs extra time.	3,50 (0,84)

1 = completely disagree, 2 = disagree, 3 = neutral, 4 = agree, 5 = completely agree, − = no opinion

**Table 2:** Scores on the TREAT State-of-Mind questionnaire (clinicians)

Subscale	Items	Mean Score (SD)
Usage behaviour	2. If it is up to me, I will start using TREAT as soon as possible.	4,17 (1,60)
	3. When TREAT becomes available I will actively use it.	4,83 (0,41)
Support	1. I express my concerns about TREAT.	3,00 (2,19)
	26. I will tell people it is good TREAT has been developed.	3,33 (1,86)
Power	4. Because of TREAT I expect to have more influence on the way I do my job.	3,50 (0,55)
	5. Because of TREAT I expect to become more dependent on others.	1,83 (0,41)
	18. I experience TREAT as a form of behavioural control.	1,50 (0,84)
Issue-impact	6. My job will remain about the same with TREAT.	3,33 (1,21)
	7. I expect TREAT to have much influence on the way I do my job.	3,00 (0,89)
	8. I expect TREAT to have much influence on the way most clinicians of the psychosis department do their job.	3,17 (1,17)
	9. I expect TREAT to have much influence on patientcare in the psychosis department.	3,50 (1,52)
Emotion	10. Use of TREAT fits with my professional values and beliefs.	3,67 (0,82)
	11. Use of TREAT fits with providing good clinical care.	4,33 (0,52)
	12. I am proud of the fact that TREAT has been developed and is being investigated.	4,17 (0,98)
	13. I am worried about the introduction of TREAT.	2,00 (1,10)
Ease of use	14. TREAT is easy to use.	4,33 (0,82)
	15. Working with TREAT requires little (extra) mental effort.	4,33 (0,52)
	20. The lay-out / arrangement of TREAT appeals to me.	3,17 (1,47)
Usefulness	16. I expect to be able to better perform my job, because of TREAT.	4,42 (0,66)
	17. I expect to receive more support in my job, because of TREAT.	3,67 (1,03)
	21. TREAT helps with the interpretation of the ROM-Phamous outcome.	4,33 (0,82)
	22. I expect TREAT to offer support in drafting the treatment plan.	4,17 (1,17)
	23. Because of TREAT I am more aware of the different treatment options that are available.	3,92 (0,67)
	27. Because of TREAT I am more aware of the purpose of ROM-Phamous.	2,67 (1,21)
Facilitating conditions	19. I expect to have enough time to use TREAT in my daily work.	4,00 (1,10)
	24. Because of TREAT I can work more efficiently.	4,08 (0,49)
	25. Using TREAT costs extra time.	2,33 (1,03)

1 = completely disagree, 2 = disagree, 3 = neutral, 4 = agree, 5 = completely agree, − = no opinion

**Table 3:** Topics mentioned in the brief open interview about TREAT (clinicians)

Positive feedback	Negative feedback
TREAT improved the efficiency of the treatment session. ^5^	The treatment recommendations were sometimes repetitive, when patients had already received certain treatment options in the past. ^1,3^
TREAT was a good reminder to talk about certain topics, which otherwise might be forgotten. ^3,5^	The specific diagnosis of the patient was not mentioned in TREAT. ^3^
The visual feedback was experienced as pleasant. ^3^	The treatment recommendations did not add much, new information. It was however convenient to explicitly go through the different options. ^4^
The visualizations were especially useful for the patient and it led to more shared-decision making. ^1^	The cut-off scores for the somatic parameters in TREAT were different than the cut-off scores the general practitioner uses. This is confusing. ^2^
Because of TREAT the discussion of the ROM results became a more explicit moment to make decisions. ^1^	The print version of TREAT was too long. The graphs take up much space. ^2^
When the treatment guidelines change, TREAT needs to be updated. The maintenance of TREAT is important. ^2^	The information the ROM nurse added to the ROM results did not appear in TREAT. Because of this, important information was sometimes missing. ^2^
ROM-Phamous was confusing and TREAT has made this better and clearer. ^6^	It is a risk that clinicians will only follow TREAT and forget about other potential problems. ^5^
Certain treatment options in the recommendations were new and I would not have thought of these options without TREAT. An example was ‘peer support groups’. ^6^	It would be helpful if TREAT could also lead to a template for a treatment plan. ^5^
The treatment session was more structured and I had the feeling we had discussed all the important issues, because of TREAT. ^5^	It would be nice to be able to compare ROM results of previous years with current results. ^1^

Clinician identifier: 1, 2, 3, 4, 5 & 6

### Feasibility of questionnaires

Both clinicians and patients completed the CDRC questionnaires. All clinicians filled out the staff version of the CDRC but some of the patients experienced difficulties with their version of the questionnaire. They remembered most of the topics discussed in the feedback sessions but some had trouble to be specific or to categorize the different topics or mention specific treatment options that were suggested.

## Discussion

A ROM inventory in 2011, for the Dutch ministry of health, welfare and sport, recommended combining ROM to CDAs to improve the treatment process within mental healthcare [25]. Despite these recommendations such systems are currently unavailable. With TREAT, a computerized CDA has been developed linking ROM to treatment guidelines. In this way TREAT generates personalized treatment advice for the treatment of patients with psychotic disorders. The primary aim of this pilot study was to describe the development of TREAT and assess its feasibility for daily clinical practice.

### Clinicians’ evaluations

In general clinicians were positive about working with TREAT. Most of them found the system easy to use without requiring extra mental effort or time. This is important as usability and limited time investment are the two most important factors affecting successful implementation of CDAs [26]. One clinician did prefer the printed TREAT report for the feedback sessions because this person felt that the computer could disturb communication with patients. However, concerns from a previous study that computer use would be distracting, decrease eye contact or depersonalize the interaction [27], were not replicated. All clinicians indicated they would like to continue working with TREAT as they found it fitting with good clinical practice. Some concerns were expressed about the recommendations being or becoming too familiar over time. Lack of novelty could potentially stop clinicians from working with the application. In contrast, some clinicians became more aware of guidelines and discovered new treatment options. There is growing evidence that treatment in accordance with guidelines within mental healthcare, can positively affect patient care [8, 28, 29]. One clinician suggested that the ROM-Phamous results of previous years should be incorporated in order to evaluate changes over time. This exemplifies that the TREAT algorithms can be improved and tailored even more to individual patients. CDAs sometimes fail to take contextual information into account or have algorithms that insufficiently fit complex patient scenarios [30]. When asking clinicians about their opinions regarding ROM-Phamous, they indicated that the outcomes are not always easy to use for clinical decision making. This is in line with previous studies showing that the outcomes of ROM-Phamous are not used to its full potential in daily clinical practice [17, 18, 31]. TREAT simplifies the interpretation of these outcomes and facilitates a basis for more explicit decision making. Future research should focus on the effects of TREAT in the clinical decision making process. Lessons learned can improve the TREAT application, but may also help to develop other CDA’s.

### Future adjustments & research

Adequately informing patients about their health and available treatment options is the future of healthcare, in which CDAs should play a pivotal role. While CDAs are commonly used in medical fields such as oncology, orthopedics and cardiology, its use in mental healthcare is still very rare. With TREAT a high quality and easy to use CDA is now available in this field. Some adjustments will be made to the system based on the results of this pilot study. For instance the algorithms will be updated to ensure that TREAT reports can still be generated when part of the data is missing. ROM-Phamous consists of multiple instruments so patients are not always able to complete all questionnaires. Furthermore, two printable versions of the TREAT report will be added, namely a summary and the complete report. These adjustments will enable a large follow-up study aiming to investigate the effects of TREAT on clinical decision making. Because there are hardly any validated methodologies available to assess the content of treatment sessions in mental healthcare, modified and translated versions of the CDRC questionnaires were tested. Clinicians were able to fill out their version. However, patients experienced difficulties in categorizing the different topics that were discussed during the treatment sessions. These difficulties probably reflect cognitive difficulties in line with their psychiatric problems. Therefore, we will use only the staff version of the CDRC questionnaire for future research. Clinicians reported that patients appreciated the TREAT report and that it could improve shared decision making. Previous research in somatic medicine supports the notion that CDAs are effective in improving shared decision-making [20]. Recently there have been calls for widespread access to CDAs to improve the level of shared decision-making within mental healthcare [32]. Patients prefer an active role in the decision-making process and are more likely to adhere to their treatment plans if they actively participated in the draft [33]. The follow-up study is designed to assess whether CDA’s, in this case TREAT, can indeed increase shared decision making within a mental healthcare setting. So far research on computerized CDAs shows that they may improve disease management and diagnostics, however the effects on patients’ functioning and final clinical outcome remains unclear [34]. Assessing the effects of TREAT on patients’ symptoms, physical health and psychosocial functioning, will be another important goal of the follow-up study. If a CDA like TREAT is beneficial in treatment of psychotic disorders, it might be worthwhile to develop similar systems for different patient groups.

### Strengths and limitations

To the best of our knowledge, this study presents the first computerized CDA combining ROM and treatment guidelines in an electronic patient record within mental health care. TREAT was developed in close collaboration with healthcare professionals. This is a strength of TREAT, as sufficient knowledge of developers about the target group of CDAs is important for successful implementation [26]. ROM is sometimes experienced as behavioural control by some users who feel obliged by political or financial motives [18]. This was not replicated in previous or the current study [17, 18], as all clinicians had positive opinions about ROM-Phamous. A limitation of the current study is the small sample of only six clinicians from the same psychiatric institute. Although large enough to test the applicability of TREAT in daily clinical practice, a larger sample of clinicians from multiple centres are preferred when testing the effects of the application on the clinical decision making process and patient outcomes.

## Conclusions

This pilot study describes the development and first evaluation of a computerized CDA (TREAT) linking ROM to clinical guidelines to generate personalized treatment recommendations in the treatment of psychotic disorders. The primary aim was to assess the feasibility of TREAT for daily clinical practice by evaluating clinicians’ opinions when working with the system. In sum, clinicians found TREAT useful for daily clinical practice, easy to use, fitting with good clinical care and all of them would like to use the system in the future. TREAT was not felt to reduce clinicians’ professional autonomy nor was it perceived as behavioural control. Clinicians expect TREAT to have a positive impact on their daily job but are unsure if it will improve patient outcomes, such as reduced symptoms and improved psychosocial wellbeing. The application will be adjusted and improved for a follow-up study based on the results from this pilot. The follow-up study will investigate the effects of TREAT on shared decision-making, the clinical decision-making process and patient outcomes.

## Additional file

Additional file 1: Clinical decision making in daily care - Staff version. (DOCX 41 kb)

## Abbreviations

CDAs: Clinical decision aids
CDRC: Clinical Decision Making in Routine Care
FACT: Functional Assertive Community Treatment
ROM: Routine outcome monitoring
ROM-Phamous: Routine outcome monitoring - pharmacotherapy monitoring and outcome survey
TREAT: TReatment-E-AssisT
