# Supplementary material for: The development and evaluation of a computerized decision aid for the treatment of psychotic disorders
Source: BMC Psychiatry. 2018 Jun 1;18:163. doi: 10.1186/s12888-018-1750-7 (PMC5984829; doi:10.1186/s12888-018-1750-7)
Supplement: Supplementary file 1 — Clinical decision making in daily care - Staff version. (DOCX 41 kb) [file 12888_2018_1750_MOESM1_ESM.docx]

**Clinical decision making in daily care - Staff version**

**(Clinical Decision-making in Routine Care – Staff (CDRC-S))***

These questions are about the consult with your patient

1. When did the consult took place? (DD.MM.YYYY)

|  |  |  |  |  |  |  |  |
| --- | --- | --- | --- | --- | --- | --- | --- |

2. When did the ROM-Phamous screening took place? (DD.MM.YYYY)

|  |  |  |  |  |  |  |  |
| --- | --- | --- | --- | --- | --- | --- | --- |

3. When did the ROM-Phamous discussion took place? (DD.MM.YYYY)

|  |  |  |  |  |  |  |  |
| --- | --- | --- | --- | --- | --- | --- | --- |

4. How long did the consult take? (min.)

|  |  |
| --- | --- |

5. Did you use TREAT for this consult?

|  |
| --- |

|  |
| --- |

Yes NO

6. If the consult was not completed in one session, please indicate when the second session took place.

|  |  |  |  |  |  |  |  |
| --- | --- | --- | --- | --- | --- | --- | --- |

|  |  |
| --- | --- |

(DD.MM.YYYY) (min.)

7. Are you the primary clinician of this patient?

YES  NO 

8. What is your occupation?

|  | Nurse-practitioner |
| --- | --- |
|  | Psychiatrist |
|  | Psychologist |
|  | Physician |
|  | Different ____________________ |

9. What were the topics about which you and your patient had to make a decision during the consult.

*Please indicate which topics were discussed and whether or not a decision was made about the treatment. Please also indicate which decision was made or why no decision was made. Keep in mind that not chancing the current situation is also a decision.*

|  | *Not discussed* | *Discussed,* ***no*** *decision* | *Discussed,* ***with*** *decision* |
| --- | --- | --- | --- |
| Positive symptoms | ☐ | ☐   - Patient wants to re-evaluate treatment options - Patient declines treatment - Different ………… | ☐   - Dose increase antipsychotic - Change antipsyschotic - Add medication to antipsychotic - rTMS - ECT - CBT - HIT - Psycho education - No changes - Different ……….. |
| Negative symptoms | ☐ | ☐   - Patient wants to re-evaluate treatment options - Patient declines treatment - Different ………… | ☐   - Dose increase antipsychotic - Change antipsychotic - Add medication to antipsychotic - rTMS - ECT - CBT - Peer support groups - Music therapy - Psychomotor therapy - Stimulate activation - No changes - Different ……….. |
|  | *Not discussed* | *Discussed,* ***no*** *decision* | *Discussed,* ***with*** *decision* |
| Depressive symptoms | ☐ | ☐   - Patient wants to re-evaluate treatment options - Patient declines treatment - Different ………… | ☐   - Specific treatment depression - Lower dose antipsychotic - Change antipsychotic - Add antidepressant to antipsychotic - No changes - Different ……….. |
| OCD | ☐ | ☐   - Patient wants to re-evaluate treatment options - Patient declines treatment - Different ………… | ☐   - Specific treatment for OCD - No changes - Different ……….. |
| Substance abuse | ☐ | ☐   - Patient wants to re-evaluate treatment options - Patient declines treatment - Different ………… | ☐   - Specific treatment substance abuse - Add clozapine - No changes - Different ……….. |
| Aggression | ☐ | ☐   - Patient wants to re-evaluate treatment options - Patient declines treatment - Different ………… | ☐   - Add Clozapine - Psychomotor therapy - No change - Different ……….. |
|  | *Not discussed* | *Discussed,* ***no*** *decision* | *Discussed,* ***with*** *decision* |
| Anxiety | ☐ | ☐   - Patient wants to re-evaluate treatment options - Patient declines treatment - Different ………… | ☐   - Specific treatment anxiety - Change dose antipsychotic - No change - Different ……….. |
| Social relationships | ☐ | ☐   - Patient wants to re-evaluate treatment options - Patient declines treatment - Different ………… | ☐   - Peer support groups - Stimulate activation - No change - Different ……….. |
| Intimate relationships | ☐ | ☐   - Patient wants to re-evaluate treatment options - Patient declines treatment - Different ………… | ☐   - Peer support groups - No change - Different ……….. |
| Relationship with family | ☐ | ☐   - Patient wants to re-evaluate treatment options - Patient declines treatment - Different ………… | ☐   - Use Triade map - No change - Different ……….. |
| Sexuality | ☐ | ☐   - Patient wants to re-evaluate treatment options - Patient declines treatment - Different ………… | ☐   - Lower dose antipsychotic - Change antipsychotic - Add medication - No change - Different ……….. |
|  | *Not discussed* | *Discussed,* ***no*** *decision* | *Discussed,* ***with*** *decision* |
| Housing | ☐ | ☐   - Patient wants to re-evaluate treatment options - Patient declines treatment - Different ………… | ☐   - No changes - Different ……….. |
| Daly activity | ☐ | ☐   - Patient wants to re-evaluate treatment options - Patient declines treatment - Different ………… | ☐   - IRB - IPS - No changes - Different ……….. |
| Personal safety | ☐ | ☐   - Patient wants to re-evaluate treatment options - Patient declines treatment - Different ………… | ☐   - No changes - Different ……….. |
| Hypertension | ☐ | ☐   - Patient wants to re-evaluate treatment options - Patient declines treatment - Different ………… | ☐   - Lifestyle advice - Reference to physician - No changes - Different ……….. |
| (Pre)diabetes II | ☐ | ☐   - Patient wants to re-evaluate treatment options - Patient declines treatment - Different ………… | ☐   - Lifestyle advice - Reference to physician - No changes - Different ……….. |
|  | *Not discussed* | *Discussed,* ***no*** *decision* | *Discussed,* ***with*** *decision* |
| Dyslipidemia | ☐ | ☐   - Patient wants to re-evaluate treatment options - Patient declines treatment - Different ………… | ☐   - Lifestyle advice - Reference to physician - No changes - Different ……….. |
| Weight | ☐ | ☐   - Patient wants to re-evaluate treatment options - Patient declines treatment - Different ………… | ☐   - Lifestyle advice - Psychomotor therapy - Reference to dietitian or physician - No changes - Different ……….. |
| Smoking | ☐ | ☐   - Patient wants to re-evaluate treatment options - Patient declines treatment - Different ………… | ☐   - Stop smoking course - Reference to specialist - No changes - Different ……….. |
| Movement disorder | ☐ | ☐   - Patient wants to re-evaluate treatment - Patient declines treatment - Different ………… | ☐   - Change antipsychotic - Different medication - No changes - Different ……….. |
|  | *Not discussed* | *Discussed,* ***no*** *decision* | *Discussed,* ***with*** *decision* |
| Heightened Prolactin levels | ☐ | ☐   - Patient wants to re-evaluate treatment options - Patient declines treatment - Different ………… | ☐   - Change dose antipsychotic - Quit antipsychotic - No changes - Different ……….. |
| Anti-cholinergic side-effects | ☐ | ☐   - Patient wants to re-evaluate treatment options - Patient declines treatment - Different ………… | ☐   - Lower dose antipsychotic - Change antipsychotic - Lifestyle advice - No changes - Different ……….. |
| Other discussed topics (please specify)  ________________  ________________  ________________  ________________ |  | ☐   - Patient wants to re-evaluate treatment options - Different ………… | ☐   - No changes - Different ……….. |

Thank you for completing this questionnaire.

* Deze vragenlijst is gebaseerd op de CDRC vragenlijst van de CEDAR study group**. Wij hebben de lijst naar het Nederlands vertaald en aangevuld om hem passend te maken voor het TREAT-onderzoek.

** Konrad J, Loos S, Neumann P, Zentner N, Mayer B, Slade M, Jordan H, De Rosa C, Del Vecchio V, Égerházi A, Nagy M, Krogsgaard Bording M, Østermark Sørensen H, Kawohl W, Rössler W, Puschner B (2015) *Content and implementation of clinical decisions in the routine care of people with severe mental illness*, Journal of Mental Health, **24**, 15-19.
